# Parenting in Adversity: Effects of Older Caregivers, Biological Carers and Troubled Carers on Child Outcomes in High HIV-Affected Communities

**DOI:** 10.1007/s10823-023-09482-6

**Published:** 2023-05-27

**Authors:** Lorraine Sherr, Ana Macedo, Mark Tomlinson, Sarah Skeen, Imca S. Hensels, Kathryn J. Steventon Roberts

**Affiliations:** 1 Institute for Global Health, University College London, Rowland Hill Street, London, NW3 2PF UK; 2 Institute for Life Course Health Research, Department of Global Health, Stellenbosch University, Stellenbosch, South Africa; 3 School of Nursing and Midwifery, Queens University, Belfast, UK; 4 Amsterdam Institute for Social Science Research, Faculty of Social and Behavioural Sciences, University of Amsterdam, Amsterdam, Netherlands; 5 Department of Psychology, University of Manchester, Manchester, UK; 6 Department for Social Policy and Intervention, University of Oxford, Oxford, UK

**Keywords:** Ageing, Caregiver, Child development, HIV/AIDS, Mental health, Sub-Saharan Africa

## Abstract

Caregiving by older adults is a common phenomenon, enhanced in the era of HIV infection. This longitudinal study was set up to examine the effect of caregiver age, relationship and mental wellbeing on child (4–13 years) outcomes (psychosocial and cognitive) in a sample of 808 caregiver- child dyads in South Africa and Malawi. Respondents were drawn from consecutive attenders at Community Based Organisations (CBOs) and interviewed with standardised inventories at baseline and followed up 12–15 months later. Analysis focused on three separate aspects of the caregiver; age, relationship to the child, and mental wellbeing, results are stratified with regard to these factors. Results showed that compared to younger caregivers, over 50 years were carrying a heavy load of childcare, but caregiver age for the most part was not associated with child outcomes. Being biologically related to the child (such as biological grandparenting) was also not a significant factor in child outcomes measured. However, irrespective of age and relationship, caregiver mental health was associated with differences in child outcome – those children of caregivers with a greater mental health burden were found to report experiencing more physical and psychologically violent discipline. Over time, the use of violent discipline was found to reduce. These data suggest that older caregivers and grandparents are providing comparable care to younger caregivers, for young children in the face of the HIV epidemic and that interventions should focus on mental health support for all caregivers, irrespective of age or relationship to the child.

## Introduction

Caregiving is a challenge – especially in the era of HIV infection where families experience multiple burdens while shouldering illness (Knight & Yamin, 2015). Much has been written about alternative care arrangements for children affected by parental HIV and AIDS (Mokgatle & Madiba, 2015), as well as the needs of caregivers in the presence of HIV (Kidman & Thurman, 2014). As HIV can affect and infect multiple family members, there is a considerable caregiving burden in these challenging circumstances (Skeen et al., 2014). Not only are caregivers faced with HIV infected or exposed children, but they will be looking at a number of additional burdens as a result of the HIV epidemic. They themselves may be bereaved, HIV-positive or ill, and it is not unlikely that there will be multiple individuals in the family with HIV. The bedfellows of HIV infection and illness also directly affect caregiving, such as poverty, unemployment, food insecurity (Garcia et al., 2013), relationship strain, secrecy (Hejoaka, 2016), bereavement, mental ill health and economic challenges as assets are diverted to HIV care requirements while replenishment is constrained by illness and unemployment (Stein et al., 2014). The literature has shown how the elderly have been part of the caregiving response (Pandit & Vishnuravardhan, 2014; Bohman et al., 2009; Schatz & Seeley, 2013), called in to provide child care in the presence of parental illness or death (Kasedde et al., 2014; Munthree & Maharaj, 2010). Parenting behavior in resource poor settings among older caregivers of children orphaned from AIDS is scarce – with a study in Kenya showing aggressive parenting and high stress, but with no corresponding child outcome data (Oburu & Palmerus, 2003).However, within the extended family systems operating in many cultures, the elderly may always have been involved in caregiving (Mugisha et al., 2013) and it is unclear whether the HIV epidemic has altered this in any substantial way (Mokgatle & Madiba, 2015). When parents die, alternative care arrangements may be vested in the grandparents, the wider family or with designated caregivers who are not directly related to the child either as a formal or informal arrangement. There is an emerging literature documenting the stress associated with such caregiving (Ice et al., 2015) with a number of additional burdens described (Govender et al., 2012; Ogunmefun et al., 2011). These can affect both the caregiver and the developmental trajectory and quality of care for the child (Punpachi et al., 2012).

Grandparents are often the ones to take on the responsibility of caregiving (Hadfield, 2014), with interesting ramifications on their health and wellbeing as well (Ice et al., 2015; Ogunmefun et al., 2011). Such caregiving may not have been anticipated at a time when the elderly expected retirement or care from their own children. HIV-associated circumstances also bring additional challenges such as stigma within the community (Messer et al., 2010; Ogunmefun et al., 2011; Singh et al., 2011; Surkan et al., 2010), or health needs of HIV-infected or -affected children (Balew et al., 2010), often with little support (Boon et al., 2009) The nature of the relationship of the child to the caregiver may be important in determining quality of caregiving and subsequent developmental progress (Ricter et al., 2017; Rutakumwa et al., 2015). A study in China showed that for double orphans, children in the care of biological grandparents scored better on all psychological measures when compared to children in the care of non-relative carers (Zhao et al., 2010). Similar data is not readily available in Africa. Caregiver mental health has also been found to be associated with child development outcomes, Bennett et al. (2016) identified poor mental health to be associated with long term worse child outcomes across psychosocial domains, growth and cognition. Many studies focus on parental mental health, with other groups such as grandparents or other kin often excluded from the literature. There also remain a dearth of literature investigating this association within sub Saharan Africa, where burdens such as poverty and HIV often result in alternatives to sole parental care of children..Caregiver factors such as age, relationship or personality may all interact (Mhaka-Mutepfa et al., 2014). The child development and parenting literature suggests that the quality of caregiving and parenting is an important determinant of child outcome (Branjerdporn et al., 2017) – perhaps irrespective of the source. In the HIV arena, family structure has been explored in relation to HIV-specific outcomes (Thielman et al., 2012) such as ART adherence(Skovdal et al., 2011) and sexual risk behaviours among the older children (Pilgrim et al., 2014), as well as economic security (Acharya et al., 2013) and poverty ramifications (Tamasane & Head, 2010). Caregiving from wider community members is often studied in terms of institutions, with few studies outside of boarder sociological and anthropological investigations exploring community-based experiences.

This paper thus provides an exploration of caregiver factors which may affect child development outcome. Belsky (1984) provides for an ecological framework on child development that includes the role of broader social, parental and child factors (Belsky, 1984). Using this framework, quality care for children is seen as fundamental to development. The landscape of care in areas of high HIV burden has seemingly resulted in alterationqualityregiving response, with an emphasis on older caregivers. Yet, there remains a dearth of literature regarding the impact of factors affecting caregivers that may impact on child outcomes, particularly from community level data. As outlined above, caregiver age as well as relationship to the child, duration of care and wellbeing may have implications for child development. This longitudinal study aims to document the experience of caregiving providing comparisons for both caregiver and child outcomes, according to age of the caregiver, relationship to the child, and caregiver mental health. Specific objectives are to explore similarities and differences in the characteristics of the caregivers in the sample by age (older > 50 years and younger < 50 years), examine child outcomes stratified by caregiver age and to explore child outcomes over time by caregiver age (inclusive of wellbeing, behavior, cognition, and violence exposure) Further analysis exploring the impact of caregiver-child relationship status, duration of care and caregiver mental health on these outcomes will also be undertaken. The study is based in South Africa and Malawi, two countries with high HIV burden and a history of HIV and AIDS mortality as well as poverty.

## Method

### Procedure

Caregiver-child dyads were recruited from community-based organisations (CBOs) in South Africa and Malawi. All 11 study funding partners (Comic Relief, UNICEF, REPSSI, World Vision, Bernard van Leer Association, Firelight Foundation, AIDS Alliance, Stop AIDS Now) provided a list of funded CBOs in the two regions (*n* = 558). Using a computer-generated random sampling frame, stratified by geographic location and funder, 28 CBOs were generated (24 in South Africa and 4 in Malawi). All 28 CBOs approached who met the inclusion criteria (non-government funding, still operational and providing services to children infected or affected by HIV) agreed to participate. Trained researchers visited the CBOs and for the duration of the visit recruited consecutive child attendees, aged 4–13 years together, with their primary caregiver. Primary caregivers were defined as those individuals who cared for the child more than 4 days in a week and took major responsibility for the child. Informed consent was provided by both the caregivers and assent from the children. The interviews were conducted with child and adult separately at baseline (2011–2012) and repeated measures were gathered at follow-up 12 to 15 months later (2013–2014). Refusal rate was low (0.7%) at baseline. At follow-up, 86.5% of child-carer dyads were reached. The study was passed by University College London and University of Stellenbosch Ethical boards (reference number 1478/002 and N10/04/112 respectively). Questionnaires were constructed to explore caregiver and child psychosocial functioning, together with background demographic factors.

### Materials

#### Caregiver Measures

All caregivers provided information on age, biological sex, living circumstances (inclusive of household employment, type of home lived in), HIV status (themselves and the child), education, employment and length of time as primary caregiver. Caregivers completed the Shona Symptom Questionnaire (SSQ; α = 0.86) (Patel et al., 1997) providing data on common mental problems on a well validated regional scale and the Patient Health Questionnaire (PHQ; α = 0.89) (Kroenke et al., 2001) for depression and anxiety. Suicidal ideation was scored on a binary (yes/no) question and the multiple mental health burden variable was scored on the presence (above the cut-off for caseness) of any of the four measured above.

#### Child Measures

Fourteen measures were selected to explore child outcomes, these measures focused on variables representative of or linked to developmental outcomes inclusive of mental health and wellbeing, cognition and educational attainment, positive and negative community interactions, exposure to violence and discipline experience. All measures were selected with a focus on age appropriateness, previous use in sub-Saharan Africa, validation and ease of administration (Loxton et al., 2006; Madge et al., 1985; Skinner et al., 2014).

##### Child Wellbeing

Regarding the child measures, depression was measured using the short form of the Children’s Depression Inventory (CDI; α = 0.62) (Kovacs, 1992), which generates a score between 0 and 18, with a higher score indicating more depressive symptoms. Child trauma was measured utilising the Trauma Symptom Checklist for Children (TSCC; α = 0.74) (Briere, 1996), with a total score from 0 to 30 and higher scores indicating worse PTSD symptomatology. Child self-esteem was measured using the Rosenberg Self-Esteem Scale (α = 0.60) (Rosenberg, 1965), with a score also ranging from 0 to 30 and higher scores indicating better self-esteem. The Strengths and Difficulties Questionnaire (SDQ) (Goodman, 1997) was used to measure behavioural and emotional problems. The SDQ is divided into subscales, each including several items on a three-point Likert scale.

##### Child Cognition and Education

Cognitive measures were administered by trained data collectors utilising the Digit Span Test for working memory(Wechsler, 2004) and the Draw-a-Person Test for general cognitive abilities(Harris & Goodenough, 1963). Educational risk was measured using a composite score from caregiver rating of school enrolment, attendance, achievement, absence, and performance. Educational risk items were taken from the Child Status Index (CSI) tool (O'Donnell et al., 2013). All items are binary, resulting in a total score from 0 to 5, with a higher score indicating higher educational risk.

##### Child Community Interactions

Children’s perception of stigma was measured using five binary items with a score ranging from 0 and 5 and higher scores indicating higher perceived stigma. Children’s perception of community support was rated using four binary items with scores from 0 to 4 and higher scores indicating better community support. Both scales were taken from the UNICEF survey tool (Snider, 2006).

##### Child Violence Exposure

Child exposure to domestic and community violence used two scale items also from the UNICEF survey tool (Snider, 2006), both ranging from 0 to 8 and higher scores indicating worse exposure to violence.

##### Child Discipline Exposure

Harsh physical or psychological discipline and positive discipline scores were gathered from the caregiver-report Parent Child Conflict Tactics scale(Straus, 1995) and the ISPCAN screening tool (Runyan et al., 2009). These scales comprised two and four items respectively. Total scores could range from 0 to 8 and 0 to 16 respectively, with higher scores indicating greater exposure to harsh discipline. Positive discipline was measured using two items and total scores ranged from 0 to 8, with higher scores indicating the use of more positive discipline.

### Statistical Analysis

Analyses were carried out using IBM SPSS 22.0. Firstly, we compared baseline characteristics of caregivers aged 50 years or above to those below 50 years using chi-square tests. The following variables were analysed: country of residence, biological sex, relation to child, length of caring for child, education, employment, HIV status, and mental health burden. Secondly, we explored differences in the characteristics of the children (i.e., age, biological sex, parental bereavement, and HIV status) according to the caregiver age using chi-square tests. Lastly, we used a series of repeated measures ANOVA analyses to test the progression of child outcomes from baseline to follow-up according to caregiver age. Child outcomes included: mental health (depressive and trauma symptoms, self-esteem, emotional and behavioural problems), education and cognitive development (performance at school and performance on two standardised cognitive tests), child experiences of stigma and community support, exposure to violence (at home in the community), experience of harsh physical and psychological punishment, and of positive discipline. In addition, we explored progression of these 14 child outcomes over time according to the relationship of the caregiver to the child, whether the caregiver had cared for the child since birth, and whether the caregiver was suffering from mental health problems.

## Results

In this analysis, we excluded caregivers who did not participate in the follow-up interview and duplicate caregivers (i.e., caregivers who were interviewed for more that one of their children either at baseline or follow-up). Overall, 808 caregivers and their children were followed-up over a 15 month period. The mean age of caregivers at baseline was 44.17 years (SD = 14.94, range = 17–87 years) and the mean age at follow-up was 45.39 years (SD = 15.44, range = 16–89 years).

## Caregiver Age

### Baseline Caregiver Characteristics Stratified by Age

Table 1 sets out baseline comparisons between the older caregivers (above 50 years of age, *n* = 522) and the younger caregivers (below 50 years of age, *n* = 286). The distribution did not differ according to country (Malawi and South Africa) and thus the data is pooled for the remainder of the analysis. Similarly, caregiver biological sex did not differ significantly by age. The majority of caregivers were female (95.0%), and there was a small proportion of male primary caregivers in both age groups. There were significantly more biological parents in the under 50 age group (66.5% versus 9.1%), whilst there were many more grandparents in the over 50 age group (73.1% versus 4.8%; χ^2^(6) = 496.47, *p* < 0.001). Two thirds of caregivers (66.8%) had cared for the child since birth, and there was a significant variation by age with not having cared for the child since birth being much more likely in the older age group (49.3% versus 25.9%; χ^2^(1) = 45.13, *p* < 0.001). The vast majority of caregivers (66.8%) had not reached final secondary school examinations in their education. There was a significant effect of age on educational achievements, as the younger caregivers generally had a higher education level; older caregivers were more likely to have no education or completed only primary education (χ^2^(6) = 92.25, *p* < 0.001). Younger caregivers were significantly more likely to be employed (χ^2^(1) = 8.58, *p* = 0.003). Younger caregivers were significantly more likely to be HIVpositive (χ^2^(1) = 40.88, *p* < 0.001).

**Table 1:** Caregiver characteristics at baseline by age (*N* = 808)

	Overall sample (*n* = 808)	Age of caregiver < 50 years (*n* = 522)	Age of caregiver ≥ 50 years (*n* = 286)	Χ^2^ (*p*-value)
Country				2.35 (0.13)
South Africa	664 (82.2%)	421 (80.7%)	243 (85.0%)	
Malawi	144 (17.8%)	101 (19.3%)	43 (15.0%)	
Biological sex				1.99 (0.16)
Female	768 (95.0%)	492 (94.3%)	276 (96.5%)	
Male	40 (5.0%)	30 (5.7%)	10 (3.5%)	
Relation to child				**496.47 (< 0.001)**
Biological parent	373 (46.2%)	347 (66.5%)	26 (9.1%)	
Foster parent	50 (6.2%)	16 (3.1%)	34 (11.9%)	
Grandparent	234 (29.0%)	25 (4.8%)	209 (73.1%)	
Aunt or uncle	94 (11.6%)	81 (15.5%)	13 (4.5%)	
Other relative	25 (3.1%)	22 (4.2%)	3 (1.0%)	
Other	32 (3.9%)	31 (5.9%)	1 (0.3%)	
Cared for child how long				**45.13 (< 0.001)**
Since birth	532 (65.8%)	387 (74.1%)	145 (50.7%)	
Other	276 (34.2%)	135 (25.9%)	141 (49.3%)	
Education				**92.25 (< 0.001)**
None	143 (17.7%)	60 (11.5%)	83 (29.0%)	
Some primary	255 (31.6%)	140 (26.8%)	115 (40.2%)	
Primary completed	129 (16.0%)	85 (16.3%)	44 (15.4%)	
Grade 10 (std 8)	174 (21.5%)	143 (27.4%)	31 (10.8%)	
Matric	96 (11.9%)	83 (15.9%)	13 (4.5%)	
Some tertiary	7 (0.9%)	7 (1.3%)	0 (0.0%)	
Tertiary completed	4 (0.5%)	4 (0.8%)	0 (0.0%)	
Employment				**8.58 (0.003)**
Working	132 (16.3%)	100 (19.2%)	32 (11.2%)	
Not working	676 (83.7%)	422 (80.8%)	254 (88.8%)	
HIV status				**40.88 (< 0.001)**
HIV-positive	160 (19.8%)	138 (26.4%)	22 (7.7%)	
Non-HIV-positive	648 (80.2%)	384 (73.6%)	264 (92.3%)	

Bolded χ^2^ statistics and *p*-values differ significantly according to age (*p* < 0.05)

### Baseline Caregiver Mental Health Outcomes Stratified by Caregiver Age

Table 2 sets out the mental health findings for the two different age bands. Overall, 27.8% of the sample was at risk of common mental health conditions according to the SSQ. The burden was equally shared by age (χ^2^(1) = 1.09, *p* = 0.30). 11.9% of the sample scored above the cut-off for depression (moderate to severe) according to the PHQ diagnostic inventory. Age was not a significant factor in this distribution (χ^2^(1) = 0.59, *p* = 0.44). The findings show that 12.6% of caregivers reported suicidal ideation in the preceding two weeks, yet this also did not differ significantly by age (χ^2^(1) = 0.01, *p* = 0.94). In summary, the data suggest that although there is quite considerable mental health burden in this sample of caregivers, age is not a significant factor in any of these measures.

**Table 2:** Mental health of caregivers at baseline by age (*N* = 808). Data are N (%)

	Overall sample (*n* = 808)	Age of caregiver < 50 years (*n* = 522)	Age of caregiver ≥ 50 years (*n* = 286)	Χ^2^ (*p*-value)
Common mental health problems				1.09 (0.30)
SSQ < cut-off	583 (72.2%)	383 (73.4%)	200 (69.9%)	
SSQ > cut-off	225 (27.8%)	139 (26.6%)	86 (30.1%)	
Depression				0.59 (0.44)
PHQ < cut-off	713 (88.2%)	464 (88.9%)	249 (87.1%)	
PHQ > cut-off	95 (11.8%)	58 (11.1%)	37 (12.9%)	
Anxiety disorder (panic attack)				1.14 (0.29)
PHQ-PD < cut-off	734 (90.8%)	470 (90.0%)	264 (92.3%)	
PHQ-PD > cut-off	74 (9.2%)	52 (10.0%)	22 (7.7%)	
Suicidal ideation				0.01 (0.94)
No	711 (88.0%)	459 (87.9%)	252 (88.1%)	
Yes	97 (12.0%)	63 (12.1%)	34 (11.9%)	
Any MH problem				0.42 (0.52)
None	526 (65.1%)	344 (65.9%)	182 (63.6%)	
Any (4 measures)	282 (34.9%)	178 (34.1%)	104 (36.4%)	
Multiple burden				5.22 (0.27)
None	526 (65.1%)	344 (65.9%)	182 (63.6%)	
One (out of 4)	155 (19.2%)	98 (18.8%)	57 (19.9%)	
Two (out of 4)	65 (8.0%)	37 (7.1%)	28 (9.8%)	
Three (out of 4)	42 (5.2%)	32 (6.1%)	10 (3.5%)	
Four (out of 4)	20 (2.5%)	11 (2.1%)	9 (3.1%)	

### Baseline Child Characteristics Stratified by Caregiver Age

Table 3 sets out the findings for child outcomes at baseline according to the caregiver age. Older caregivers were more likely to be caring for older children (χ^2^(1) = 15.62, *p* < 0.001) and no biological sex differences between the two groups of children were identified (χ^2^(1) = 0.61, *p* = 0.43). Older caregivers were more likely to be caring for children who had lost one or both parents (χ^2^(4) = 109.28, *p* < 0.001), there were no differences by caregiver age on the HIV status of the child (χ^2^(1) = 1.09, *p* = 0.30).

**Table 3:** Children’s characteristics at baseline by caregiver’s age (*N* = 808)

	Overall sample (*n* = 763)	Age of caregiver < 50 years (*n* = 474)	Age of caregiver ≥ 50 years (*n* = 287)	χ^2^ (*p*-value)
Age				**15.62 (< 0.001)**
4–9 years	411 (51.0%)	292 (56.2%)	228 (43.8%)	
10–14 years	395 (49.0%)	119 (41.6%)	197 (58.4%)	
Biological sex				0.61 (0.43)
Boy	389 (48.1%)	246 (47.1%)	143 (50.0%)	
Girl	419 (51.9%)	276 (52.9%)	143 (50.0%)	
Parental bereavement				**109.28 (< 0.001)**
Non-orphan	309 (44.1%)	248 (56.0%)	61 (23.6%)	
Maternal orphan	123 (17.5%)	49 (11.1%)	74 (28.7%)	
Paternal orphan	130 (18.5%)	93 (21.0%)	37 (14.3%)	
Double orphan	129 (18.4%)	50 (11.3%)	79 (30.6%)	
Unknown	10 (1.4%)	3 (0.7%)	7 (2.7%)	
HIV status				1.09 (0.30)
HIV-positive	124 (15.3%)	49 (17.1%)	75 (14.4%)	
HIV-negative or unknown	684 (84.7%)	237 (82.9%)	447 (85.6%)	

Bolded *p*-values suggest a difference according to group (*p* < 0.05).

### Child Functioning Overtime Stratified by Caregiver Age

Table 4 shows child functioning outcomes over time according to the caregiver age. It is of note that across the 14 tests carried out, there were no significant differences in child outcome according to caregiver age on 13 variables (including depressive and trauma symptoms, self-esteem, educational risk, cognitive development, community stigma and support, exposure to violence and harsh or positive discipline), a significant difference was identified with regard to child scores on the SDQ problem scale. For the SDQ problems scale (comprising internalising and externalising behavioural problems), children cared by older caregivers had significantly lower reported difficulties over time (mean score was 3.04 at baseline and dropped to 2.68 at follow-up) compared to children cared by younger caregivers (mean score increased from 2.80 at baseline to 3.03 at follow-up) (*p* = 0.02).

**Table 4:** Children psychosocial outcomes over time by caregiver’s age (*N* = 763)

	Age of caregiver < 50 years (*n* = 476)		Age of caregiver ≥ 50 years (*n* = 287)		
	Baseline	Follow-up	Baseline	Follow-up	(*p*-value)^a^
CDI (*M, SD*)	0.95 (1.50)	0.84 (1.41)	0.89 (1.33)	0.81 (1.63)	n.s
PTSD (*M, SD*)	3.82 (3.09)	4.42 (3.77)	4.12 (3.62)	4.29 (3.88)	n.s
Self Esteem (*M, SD*)	21.11 (2.86)	22.25 (3.91)	21.28 (2.80)	22.26 (3.87)	n.s
SDQ—problems scale (*M, SD*)	2.80 (2.34)	3.03 (2.56)	3.04 (2.40)	2.68 (2.46)	**0.02**
Number of educational risks (*M, SD*)	0.81 (1.06)	0.84 (1.11)	0.69 (0.76)	0.76 (1.02)	n.s
Performance on digit span (*M, SD*)	8.79 (3.76)	8.98 (3.47)	8.77 (4.16)	9.06 (3.65)	n.s
Draw-a-person test (*M, SD*)	84.13 (19.32)	90.92 (17.44)	87.04 (17.43)	92.21 (16.83)	n.s
Perceived stigma (*M, SD*)	0.55 (1.02)	0.52 (0.95)	0.48 (0.94)	0.51 (1.02)	n.s
Perceived community support (*M, SD*)	3.77 (0.59)	3.59 (0.68)	3.78 (0.60)	3.63 (0.73)	n.s
Exposure to domestic violence (*M, SD*)	1.06 (1.56)	0.84 (1.39)	1.16 (1.64)	0.89 (1.37)	n.s
Exposure to community violence (*M, SD*)	0.55 (0.87)	0.62 (0.76)	0.68 (0.94)	0.65 (0.89)	n.s
Harsh physical discipline (*M, SD*)	0.60 (0.71)	0.47 (0.67)	0.57 (0.66)	0.40 (0.64)	n.s
Harsh psychological discipline (*M, SD*)	0.81 (1.16)	0.71 (1.04)	0.81 (1.14)	0.67 (1.12)	n.s
Positive discipline (*M, SD*)	2.42 (1.05)	1.99 (1.07)	2.37 (1.05)	2.09 (1.09)	n.s

Bolded *p*-values suggest a difference according to group (*p* < 0.05)

^a^p-value is associated with repeated measures ANOVA analyses

## Caregiver Relationship to the Child

**Fig. 1:**
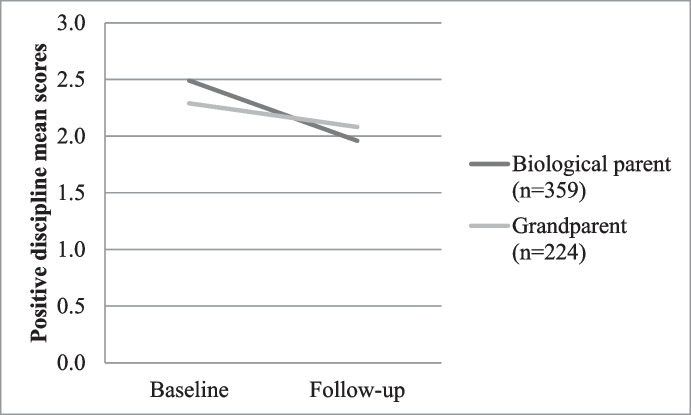
Child experience of positive discipline according to relationship to caregiver; F (1, 583) = 6.18, *p* = 0.008

**Fig. 2:**
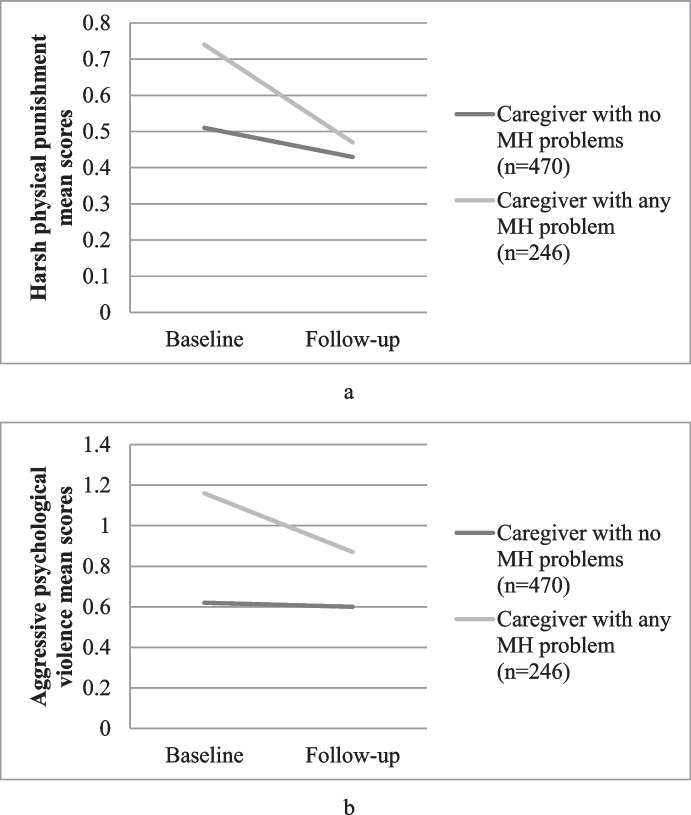
**a** Child experience of physical violence according to caregiver mental health state over time; F(1,716) = 8.74, *p* = 0.003. **b** Child experience of psychological violence according to caregiver mental health sate over time; F(1, 716) = 5.56, *p* = 0.02

The following analyses then explored whether it was the relationship to the child rather than the age of the caregiver that was a key factor in child outcome. Three groups were created for comparison, namely biological parent (*n* = 359), biological grandparent (*n* = 224) and other caregivers (*n* = 174). On all 14 variables there were no differences by group, except for positive parenting. Children cared by their biological parents experienced more positive discipline at baseline, yet the mean scores reduced slightly at follow-up. Children cared for by a biological grandparent had similar positive discipline experiences over time. The same pattern was seen when comparing only biological parents with biological grandparents (*p* = 0.008) (see Figs. 1 and 2).

## Duration of Care

Given the extended childcare systems that may well be embellished in the culture anyway, the next analysis compared the caregivers who have cared for the child since birth (*n* = 508) with those who have not cared for the child since birth (*n* = 255). Again, there were no significant differences according to length of care on any of the 14 outcome measures.

## Mental Health Status of the Caregiver

Finally, the baseline mental health status of the caregiver, irrespective of age or relationship to the child was examined. Caregivers who scored above the cut off for mental health problems on the Shona Symptom questionnaire (*n* = 470) were compared with those below the cut off and thus not exhibiting mental health problems (*n* = 246). Those with mental health problems at baseline scored significantly higher from baseline to follow up on harsh physical (*p* = 0.003) and psychological discipline (*p* = 0.02). Interestingly, for both groups, the scores reduced over time.

## Discussion

Although there is a solid literature on the growing group of older caregivers, few studies have explored in detail how this affects both caregiver and child outcomes. The findings from this study show that much of the caregiving load is carried by the over fifties – yet they do not differ in emotional burden or child outcome for the most part. It may well be argued that extended family care is common in both South Africa and Malawi and it the crucial factor may be biological relationship to the child. Again, this analysis showed that biological relationship was not associated with any of the child outcome measures, and neither was length of time caring for the child – be it from birth or later. It thus seems that for these children, caregiving is very similar for these different groups and types of caregiver relationships. It appears that grandparents are doing as good a job as biological parents. The only variable that seems to differentiate some child outcomes is related to the mental health status of the caregiver – irrespective or relationship to the child. Caregivers with mental health scores above the cut off were significantly more likely to utilise harsh physical and psychological punishments. While a causal inference cannot be drawn from this data, confounding factors should remain a consideration. The reduction in harsh punishment and child problem behavior overtime suggests a potential association between these factors and highlights the possibility of an association between mental health, child problem behavior and harsh physical punishment. The use of harsh physical punishment ameliorated for all groups over time – perhaps because of the beneficial effects of the parenting support and interventions provided by the CBO, or perhaps the ageing of the child reduced the need for such punishments. However, at follow up the difference between those with and without a mental health problem remained associated with harsh punishment of young children. It may also be possible that difficult child behavior played a role in the mental health status of the caregiver – direction of the association may need to be considered with caution.

Parenting quality has been well established as a benefit to child development and enhances multiple child outcomes (Lachman et al., 2014). There is good evidence that a number of factors associated with high HIV environments can negatively affect parenting, especially in the presence of parental physical illness and child orphanhood. The mode of effect may be through decreased parental engagement, disrupted routines, parental absence, neglect, the ramifications of poverty, stigma, isolation and grief (Armistead et al., 1995), Gillies et al., 1996, Kuo & Operario, 2011, Casale & Wild, 2012, Sipsma et al., 2013).

This study provides reassuring evidence that older caregivers are providing comparable levels of care and this is associated with comparable outcomes for children across a broad set of measures including physical, cognitive, educational, emotional, behavioural and discipline domains. It also highlights that a variety of parenting and caregiving arrangements can meet the needs of children. In terms of input, the mental health problems that caregivers face may impact their parenting style and skills and this points to potential avenues for intervention. There is a well-established literature on maternal (Murray et al., 2014; Sanger et al., 2015) and paternal depression on child outcome (Davé et al., 2008; Ramchandani et al., 2008). Our data suggest that these findings can be extended to other caregivers as well and may relate to mental health challenges beyond depression. This clearly has implications for targeted interventions by either providing care to ameliorate the underlying mental health problems in the first place, or parenting support for caregivers with mental health burden. The data cautiously suggest that Community based organisation exposure over time was associated with reduced scores on harsh psychological and physical punishment – perhaps indicating a potential delivery mechanism for such interventions.

The study has a number of limitations. Since this is an observational cohort study, no causation can be inferred from the data presented here, and the direction of effects must be considered. Moreover, all the caregivers and the children they cared for were attending CBOs, so the data here is not necessarily generalizable to the wider population without such front-line support. By confining the analysis to those with the same primary caregiver over time, those with changed caregiving arrangements – perhaps most at risk – were not accounted for in our analysis. A specific study on child development in the presence of caregiving changes may be warranted. Nevertheless, the study also has a number of strengths. The sample is large and the follow-up rate was high. Therefore, the data presented here still make an important contribution to the literature on caregiver age and child outcomes.

Our findings suggest that children of older caregivers are often equally well off as children from younger caregivers, which indicates that living with grandparents does not seem to interfere with children’s developmental potential. In the changing pattern of caregiver provision triggered by epidemics such as HIV, clear attention needs to be paid to the mental health and wellbeing of the caregiver as a key factor in determining the quality of caregiving and subsequent child outcomes. Parenting support may well be of use to all those involved in caregiving and specific support – either for parenting or for mental health problems is needed for caregivers who score above a cut off for mental health burden.
